# Short-term variability of chronic musculoskeletal pain

**DOI:** 10.3389/fpain.2025.1626589

**Published:** 2025-09-11

**Authors:** Xuanci Zheng, Swati Rajwal, Carl Ashworth, Sharon Yuen Shan Ho, Ben Seymour, Nicholas Shenker, Flavia Mancini

**Affiliations:** ^1^ Computational and Biological Learning Unit, Department of Engineering, University of Cambridge, Cambridge, United Kingdom; ^2^ Wellcome Centre for Integrative Neuroimaging, FMRIB, Nuffield Department of Clinical Neurosciences, University of Oxford, Oxford, United Kingdom; ^3^ Institute of Biomedical Engineering, University of Oxford, Oxford, United Kingdom; ^4^ Rheumatology Research Unit, Cambridge University Hospitals, Cambridge, United Kingdom; ^5^ Department of Medicine, University of Cambridge, Cambridge, United Kingdom

**Keywords:** chronic pain, pain monitoring, temporal variability, clinical severity, pain prediction

## Abstract

Chronic musculoskeletal (MSK) pain can be characterized by its temporal variability and evolution, affecting both pain management and treatment outcomes. While pain variability is traditionally studied over long timescales (e.g. days or weeks), few studies have explored short-term fluctuations (e.g. minutes to seconds) and their clinical relevance. This study investigated the short-term variability of chronic musculoskeletal pain across consecutive days, examining whether these fluctuations are stable, exhibit consistent temporal patterns, and relate to clinical severity. We also explored whether individuals with chronic MSK pain could predict their pain intensity on the following day, suggesting an ability to learn about their pain’s levels. Eighty-one participants with chronic MSK pain to the back, neck, leg or arm (22–65 years, 72% females, 28% males) rated their pain continuously over two days, using a smartphone-based app. Results indicated that pain ratings were stable and exhibited consistent temporal patterns across days, with a temporally correlated structure. High mean pain levels were associated with lower variability, possibly reflecting a stabilized pain state. Short-term pain variability negatively correlated with clinical severity, indicating that greater variability is linked to milder pain. These findings highlight the importance of short-term variability as a distinct and clinically relevant feature of chronic MSK pain, with implications for personalized pain management strategies.

## 1 Introduction

A key clinical feature of chronic ongoing pain is its temporal variability and dynamic evolution, which can complicate pain management and influence treatment decisions (1). For example, the ability to anticipate how pain will change over time can inform medication timing, pacing of activity, and psychological coping strategies (2–4). Despite its relevance, most studies have investigated pain dynamics on longer time scales, spanning days or weeks (1, 4–6). In contrast, short-term fluctuations—on the order of minutes or seconds—have received comparatively little attention, despite their potential to reveal important mechanisms of peripheral variability and endogenous pain regulation.

The transmission of endogenous noxious signals to the brain is known to follow a volatile and auto-correlated temporal structure (7–9). Continuous self-reports of spontaneous pain exhibit fractal properties, characterized by a power-law relationship between variability and the timescale of measurement. These properties vary not only between types of chronic pain (e.g., back pain vs. post-herpetic neuropathy) but also between real and imagined pain, or between spontaneous and evoked pain (8). People with chronic pain also exhibit greater variability in their ratings of experimental pain, relative to healthy controls, and such variability seems to be associated with pain catastrophizing (9).

Importantly, short-range temporal variability may reflect the functional status of pain regulatory systems. For instance, moment-to-moment pain fluctuations have been linked to activity in the brainstem in individuals with chronic neuropathic pain (10), suggesting that temporal instability may be a marker of dysregulated endogenous control. Supporting this view, medium-term pain fluctuations over 2–4 days have been shown to be more frequent and severe in individuals with high-impact temporomandibular pain compared to those with lower-impact symptoms (11). This is consistent with broader evidence of dysfunctional pain modulation in chronic pain populations (12).

Despite these findings, it remains unclear whether short-term pain variability is stable within individuals across days, and whether it reflects trait-like differences that are linked to clinical outcomes such as pain intensity, interference, or emotional distress. Understanding the consistency and clinical significance of these short-term patterns could enhance pain phenotyping and support precision medicine approaches to treatment.

We also explored whether individuals with chronic pain are capable of predicting their own pain levels for the following day. This question is grounded in both theoretical and empirical work on pain expectations, which have been shown to shape pain perception via top-down mechanisms (13–15). Expectations may also emerge from the brain’s ability to detect and learn temporal regularities in noxious input, a process referred to as temporal statistical learning (16, 17). If individuals with chronic pain can make accurate predictions about their future pain, this may reflect implicit learning of their own pain trajectories, even in the face of subjective unpredictability. Such predictive capacity could inform personalized self-management strategies and improve clinical communication.

To address these questions, we conducted a smartphone-based observational study in which participants with self-reported chronic musculoskeletal pain (experiencing pain in the back, leg, neck, or arm for more than 6 months) continuously rated their pain over two consecutive days. After completing ratings on the first day, participants also predicted the intensity of their pain for the next day and reported their confidence in this prediction. This approach allowed us to assess whether short-term pain variability is (i) consistent across days, (ii) linked to clinical features, and (iii) predictable by the individuals themselves. Findings from this study may shed light on the temporal structure and stability of chronic pain, while also identifying potential markers of pain self-awareness and regulation that could inform tailored interventions.

## 2 Methods

### 2.1 Participants and screening procedures

We recruited 200 English-speaking participants online through the Prolific platform (18), which applies an internal eligibility process excluding individuals with dyslexia, speech disorders, hearing loss, vision loss, color blindness, diabetes, respiratory disease, head injury, or coronary artery disease, as well as those who are pregnant or who had received a cancer diagnosis within the previous 12 months. As an additional step, all participants in our study completed a health screening questionnaire to further evaluate eligibility. Before the screening, all participants provided their digital informed consent, adhering to procedures approved by the Department of Engineering, Ethics Committee of the University of Cambridge. In order to ensure complete anonymity, the online questionnaires and tasks did not require participants to provide their names or contact details. The initial screening survey included a brief health questionnaire (Supplementary Material S3), focusing on medical history, along with the standard Musculoskeletal Health Questionnaire (MSK-HQ) (19). MSK-HQ (scores between 0 and 56) enables individuals with musculoskeletal conditions to report their symptoms and quality of life in a standardized way.

To be included in the experiment, participants had to meet the following criteria: (a) having provided digital consent; (b) experiencing pain in the back, leg, neck, or arm for more than 6 months; (c) having passed an attention check question; and (d) not having any neurological, psychiatric, or developmental disorders. Participants were included based on self-reported chronic pain symptoms. The screening included detailed questions about pain location, duration, and medical history, providing a reliable proxy for chronic pain conditions. This choice was deliberate, in line with our goal to study the real-world experience of individuals who identify as living with chronic musculoskeletal pain. Self-report is a widely utilized method in pain research, particularly for remote, app-based observational studies, and is supported by prior literature emphasizing the ecological validity of subjective pain assessments (20, 21). Based on these criteria, 123 participants were invited to take part in the two-day online experiment detailed in Section 2.2. Of these, 81 participants successfully completed the experiment on the first day, and 41 participants completed both days. Supplementary Figure S1 presents a flow chart illustrating the inclusion and exclusion of study participants.

Among the 81 participants who completed Day 1, there were 58 females and 23 males, with an average age of 45.6 years (±11.7), ranging from 22 to 65 years. These participants had experienced chronic pain for an average of 9.7 years (±6.7) and scored 36.6 (±9.0) on the MSK-HQ scale. Among the 41 participants who completed both days of the experiment, 32 were females and 9 were males, with an average age of 46.4 years (±11.0). These participants had been experiencing chronic pain for an average of 9.8 years (±6.6) and scored 36.2 (±10.9) on the MSK-HQ scale. Feedback from volunteers revealed that they found the task very easy to do, but not very engaging. Informal feedback from participants indicated that while the task was easy to follow, it was not particularly engaging. The relatively high dropout rate may be attributed to the online recruitment method (via Prolific) and the requirement that Day 2 participation occur at approximately the same time of day as Day 1. For analytical purposes, Day 1 data were used to address the primary research questions, while Day 2 data served to evaluate test-retest reliability.

### 2.2 Experiment protocol

The entire experiment was conducted online, on smartphones, over the course of two days. On each day, participants were prompted to engage in a continuous rating task to provide their pain intensity using an online application developed with the open-source software package PsychoPy (22). The application was hosted on Pavlovia (23). At the start of the study, participants were provided with detailed instructions, which briefly described the continuous pain rating task and a simple attention task resembling the actual procedure, and required to complete a short practice run to ensure they understood how the application worked. Once this was completed successfully, the participants continuously rated their pain for approximately five minutes on a vertical scale ranging from “Least Pain” to “Most Pain.” We chose this vertical continuous scale to facilitate intuitive and accessible real-time reporting across a wide range of devices along with minimum cognitive load during continuous rating tasks. Also, the simplicity of the anchors allowed participants to focus on moment-to-moment fluctuations rather than numerical calibration.

**Figure 1:**
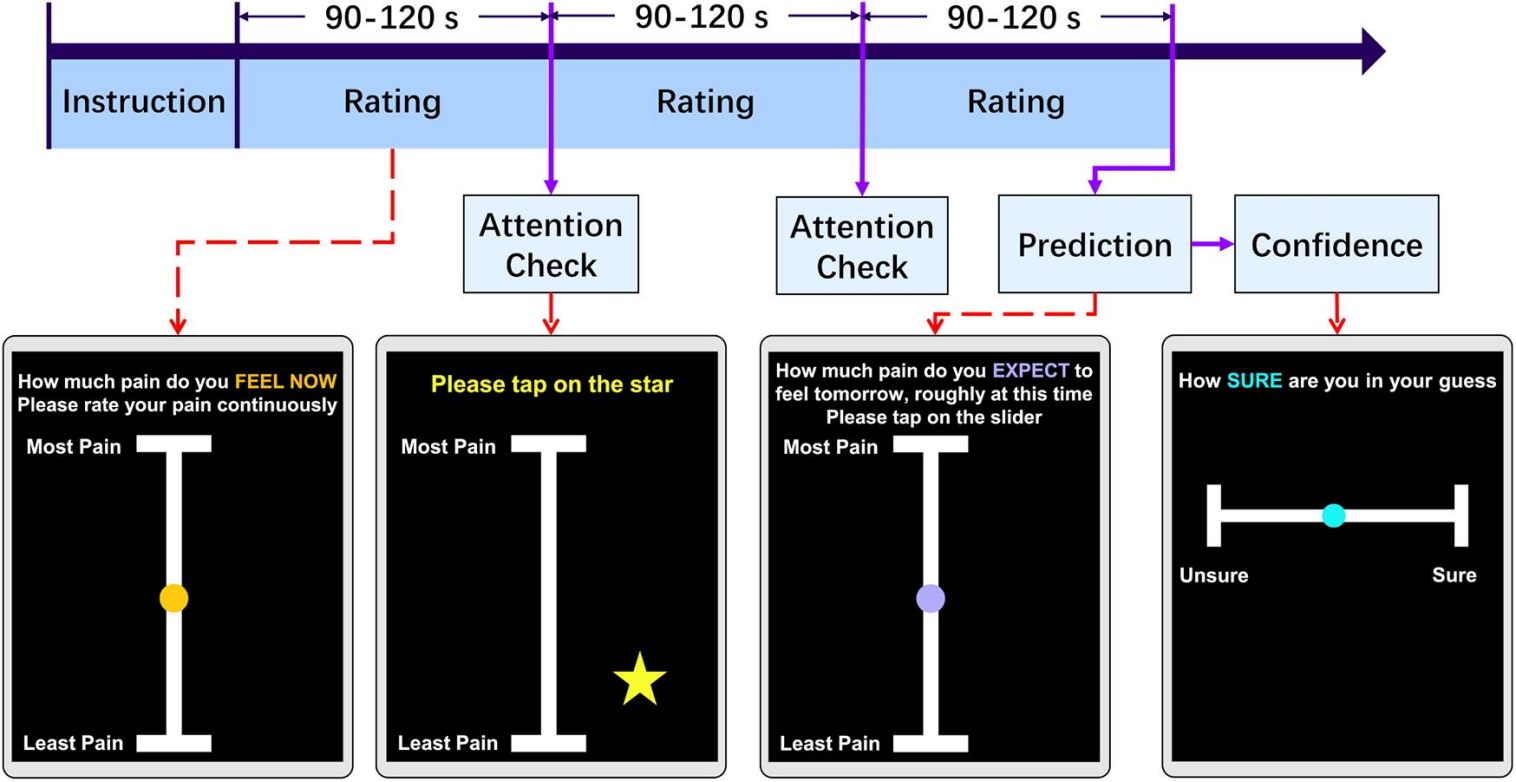
Experiment task design. Each day, participants engaged in an online pain rating task. Following instructions, volunteers were guided to continuously maintain their finger on a slider and rating their pain intensity for a randomized duration of 90 to 120 s. During the task, a randomly appearing star on the screen served as an attention check, which they tapped. This cycle repeated three times. Before finishing the task, participants were prompted to predict their pain levels at the same time the following day and express their confidence in this prediction by tapping the slider.

The interface of the application and the timeline of the experiment are depicted in Figure 1. During the experiment, two attention tests were included to assess the participants’ level of engagement with the task (tapping on a star briefly appearing on the screen), at a random time within the 90–120 s range. The attention checks interrupted the continuous rating process, which had an average sampling rate of 55 Hz, giving rise to 3 trials of variable length. Participation were considered as invalid if participants did not pass attention checks. After completing the continuous pain rating, participants were asked to estimate how much they expected their pain level to be the next day, at a similar time of day, using a visual analog scale from “Least Pain” to “Most Pain.” Although the VAS scale’s may have non-linear properties (24, 25), minor differences between paper and mobile assessments are not clinically significant (26). Participants also indicated their level of confidence in this prediction on a scale from “Unsure” (low confidence) to “Sure” (high confidence). This confidence scale was designed for simplicity, compactness and ease of use [e.g., (27)].

Finally, participants were asked to complete clinical questionnaires to provide a more complete description of their symptoms. Specifically, on day 1 of the experiment, participants completed the Brief Pain Inventory (BPI) (28), while on Day 2 of the experiment, they completed the Pain Catastrophizing Scale (PCS) (29). Answering these questions was optional. BPI helps quantifying both pain severity and functional interference across musculoskeletal and other chronic pain conditions. It is brief, validated in dozens of languages, and highly responsive to change. PCS assess the extent to which individuals tend to catastrophize, or excessively dwell on and magnify, their pain experience. Together, the BPI and PCS gave complementary snapshots- what the pain feels like and how the participant thinks about it. These are essential for interpreting the clinical significance of the high-resolution temporal data. The two questionnaires were spread over two days to avoid participants from feeling overwhelmed by the volume of questions if they were presented all at once.

### 2.3 Data pre-processing

Firstly, we removed trials which were interrupted by the participants. Given that some participants did not keep their fingers on the screen for the entire duration of each rating, causing significant data “gaps” (lack of rating data) within a trial, we segmented the rating data into epochs based on the presence of large gaps (more than 10 s). If any epoch had gaps for less than 10 s, the missing data were linearly interpolated. Epochs with more than 3 gaps that exceeded 10 s were deemed invalid and excluded. The total exclusion rate of trials was 23.8%. Following data segmentation, all ratings were resampled at a 40 Hz sampling rate for consistency.

### 2.4 Analysis of variability and reliability of continuous pain rating

To describe the variability in the continuous ratings of the participants, we calculated for each day the mean, coefficient of variation (CV) (Equation 1), and interquartile range (IQR) (Equation 2), respectively, which indicate the intensity, variability relative to the mean intensity, and dispersion of the rating of each participant. For a full day’s rating by a participant, SD represents the standard deviation of the rating; Mean is the mean value of the rating; Q1 corresponds to the first quartile (25th percentile) of the data; Q3 corresponds to the third quartile (75th percentile) of the data:

(1) $$\text{CV}=\left(\frac{\mathrm{SD}}{\mathrm{Mean}}\right)\times 100\%$$

(2) IQR=Q3−Q1

To assess the distribution of variability parameters, we generated plots for each parameter and observed that they displayed a skewed distribution. To reduce the influence of extreme values and prevent the generation of invalid results in standard statistical analyses, we applied a log transformation to reduce the skewness. Supplementary Figure S2 shows the distribution of both the raw factors and the log-transformed data. The resulting distribution showed a clearer central tendency with a reduction in the spread of values.

We evaluated the test-retest reliability of the ratings on Day 1 and Day 2, by calculating the intra-class correlation (ICC) and Pearson’s correlation, corrected for multiple comparisons using a Benjamini & Hochberg correction on the False Discovery Rate (FDR). This analysis was performed to evaluate the consistency of the above variability factors obtained from the ratings of the same participant on both Day 1 and Day 2. Furthermore, we estimated the lagged autocorrelation among the pain ratings, to determine whether pain ratings at each time point were based on recent pain levels.

To investigate the clinical significance of our variability measures (CV, IQR), we correlated them with the clinical questionnaire scores (MSK-HQ, BPI severity, and PCS). The BPI severity score (out of 10) is calculated by the scores for Questions 2, 3, 4 and 5 and then dividing by 4. For consistency, we also correlated the mean pain level with MSK-HQ, BPI severity, and PCS scores. Again, the FDR was corrected for multiple comparisons. Correlations for day 1 were calculated based on participants who attended day 1, while analyses for day 2 were performed using participants who attended both days. All statistical tests were two-tailed, and statistical significance was defined as corrected p<0.05.

### 2.5 Pain prediction

After assessing the variability of the ratings, we focused on evaluating the accuracy of each participant’s pain prediction. At the end of their participation in their task on Day 1, participants were asked to predict their intensity of pain at a similar time on the following day, along with their confidence in that prediction. To gauge the precision of their predictions, we calculated the Root Mean Square Error (RMSE) between their actual pain rating values on day 2 and the predictions they made the previous day. A higher RMSE indicates a lower prediction accuracy. To quantify their prediction accuracy, we subtracted the RMSE from the highest value on the prediction slider (10). In the following Equation 3, $R_{i}$ represents the rating data on Day 2. Pred refers to the pain prediction for Day 2 made on Day 1.

(3) $$\text{Acc}=10-\sqrt{\frac{\sum_{i=0}^{T}(R_{i}-\mathrm{Pred}{)}^{2}}{T}}$$

We correlated the prediction performance of participants (prediction and accuracy) with the mean and variability measures (CV and IQR).

## 3 Results

### 3.1 Variability and reliability of pain ratings

The mean level of rated pain, its variability (CV) and spread (IQR) were fairly stable across the two days of testing (see Table 1).

**Table 1:** Test-retest reliability of two-day participation. Mean, CV and IQR have been calculated from ratings in Day 1 and 2. Intraclass Correlation Coefficient (ICC) and Pearson’s R were calculated between factors on Day 1 in participants who attended both days and corresponding to the same participant on Day 2.

Factor	ICC	p-value (ICC)	Pearson’s R	p-value (Pearson’s R)
Mean	0.863	<0.001	0.823	<0.001
CV	0.805	<0.001	0.754	<0.001
IQR	0.788	<0.001	0.733	<0.001

To assess the comparability between participants who completed both days of the study and those who only attended the first day, we conducted Mann-Whitney U tests on the Day 1 ratings. Specifically, we compared the mean, CV, and IQR between the two groups. The results indicated no significant differences across these measures (mean: p = 0.095; CV: p = 0.788; IQR: p = 0.155), suggesting that the participants who completed only one day were not systematically different from those who completed both. This supports the inclusion of data from both groups in subsequent analyses.

To investigate the temporal dependencies in pain rating, we conducted autocorrelation analyses. First, we computed the autocorrelation across all lags for each participant’s individual ratings. These values were then aggregated across participants to calculate the mean and variance at each lag, providing a group-level summary of the autocorrelation structure on both days. Second, we calculated the lag-1 second autocorrelation, which measures the correlation between rating points separated by a 1 s interval, capturing short-term dependencies over a behaviorally meaningful timescale.

**Figure 2:**
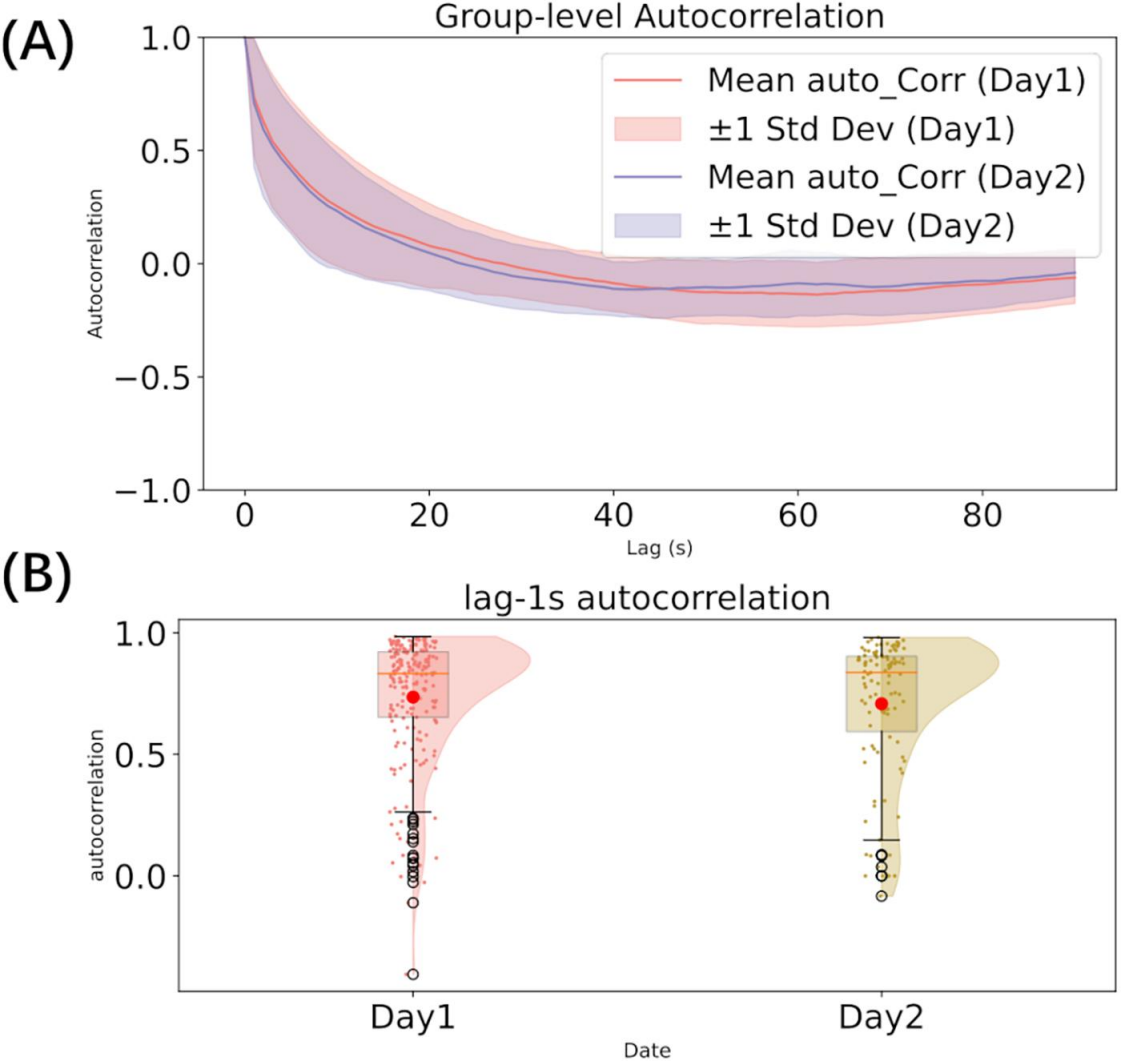
Autocorrelation analysis (A–B). **(A)** autocorrelation across all lags; **(B)** The distribution of lag-1s autocorrelation.

The visualization of the mean autocorrelation function across participants revealed a gradual decay across both days. The lag-1s autocorrelation indicated a high level of short-term dependency in the ratings (mean: 0.736 for Day 1, 0.709 for Day 2), with no significant difference between the two days (Mann-Whitney U test: U = 10855.0, p = 0.535). The group-level autocorrelation across all lags is presented in Figure 2A, and the distribution of lag-1 second autocorrelation is shown in Figure 2B.

**Figure 3:**
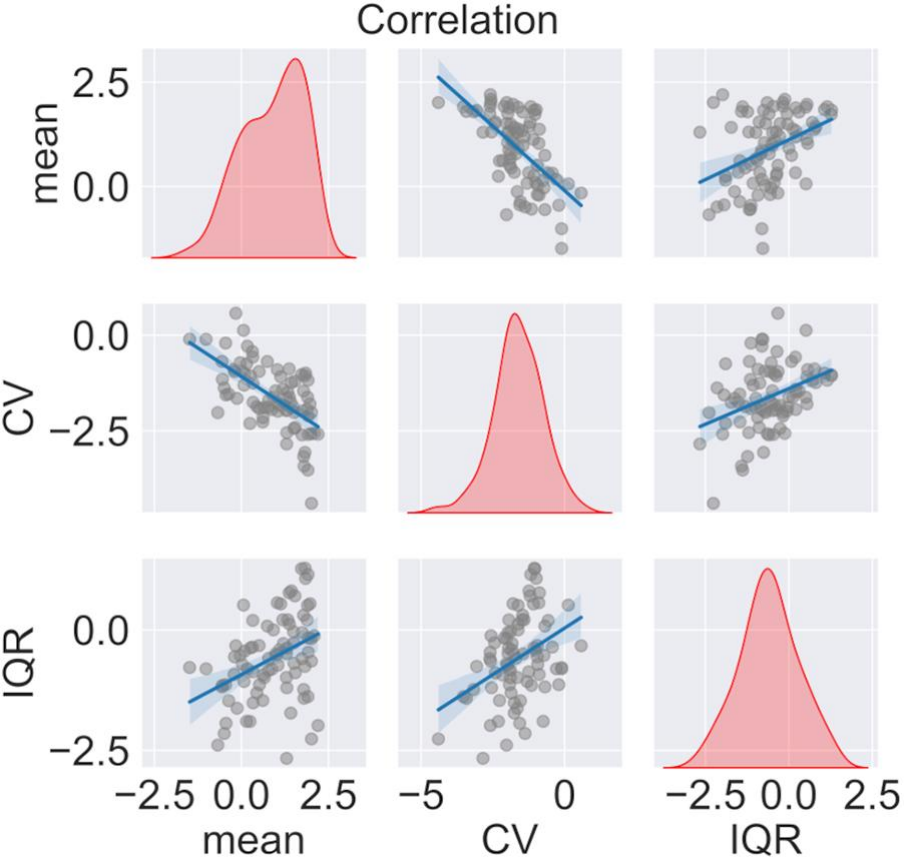
Correlation among variability parameters (mean, CV, and IQR) on Day 1. Kernel density estimation of each factor and regression of each correlation.

Mean pain levels were negatively correlated with CV (day 1: Pearson’s R = −0.608, corrected p < 0.001; day 2: R = −0.741, p < 0.001) and positively correlated with IQR (day 1: R = 0.382, p < 0.001, Figure 3; day 2: R = 0.398, p = 0.013). Hence, the more severe the intensity of the rated pain, the less variable it was, the values regressing toward their central ranges. Furthermore, as the relative variability increased, so did the data spread, but only on day 1 (CV and IQR correlation in day 1: R = 0.379, p < 0.001; day 2: R = 0.103, p = 0.435).

### 3.2 Clinical significance

**Figure 4:**
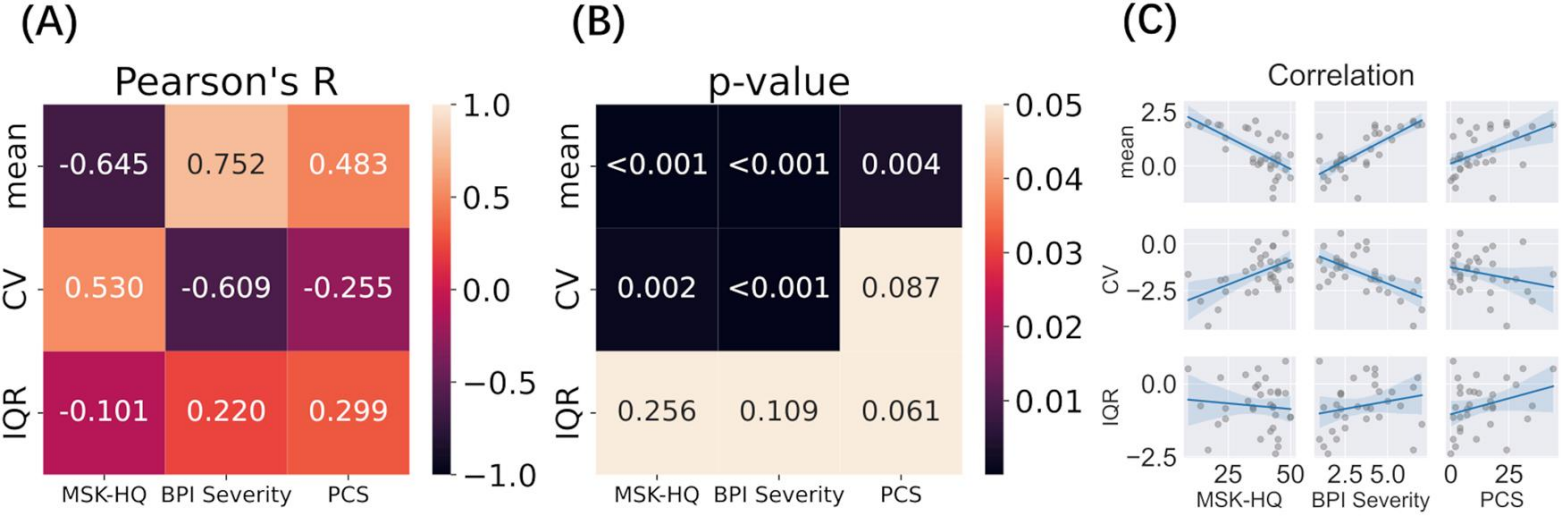
Correlation between variability measures (Day 1) and clinical outcomes (A–C). Log transformed Mean, CV, and IQR were correlated with MSK-HQ, BPI Severity, and PCS questionnaire scores. **(A)** Pearson’s correlation between variability factors and clinical outcome; **(B)** P-value of each correlation after multiple comparison; **(C)** Scatterplots and regression lines for each pairwise relationship.

Mean pain levels and variability measures (CV, IQR) on day 1 were correlated with clinical scores to explore their clinical significance (Figure 4, Supplementary Table S1). Detailed clinical information for all 81 participants is provided in Supplementary Material S4. Because the BPI was assessed on Day 1 and the PCS on Day 2, we used Day 1 data only from participants who attended both days. We found a strong negative correlation between pain variability (CV) and BPI severity and a strong positive correlation with the MSK-HQ score (n.b., the better the MSK-HQ score, the better the health status). This again indicates that the milder the pain condition, the higher its variability. The mean pain value also had a strong negative correlation with MSK-HQ and positive correlations with BPI severity and PCS.

To validate these findings, the same correlation analysis was performed on day 2 data, which showed similar results (S1): CV was strongly correlated with BPI Severity and moderately correlated with MSK-HQ, while mean pain was strongly correlated with MSK-HQ, BPI Severity, and PCS. Furthermore, the spread of pain ratings around mean values (IQR) showed a moderate positive correlation with BPI severity and PCS on day 2.

### 3.3 Pain prediction

**Figure 5:**
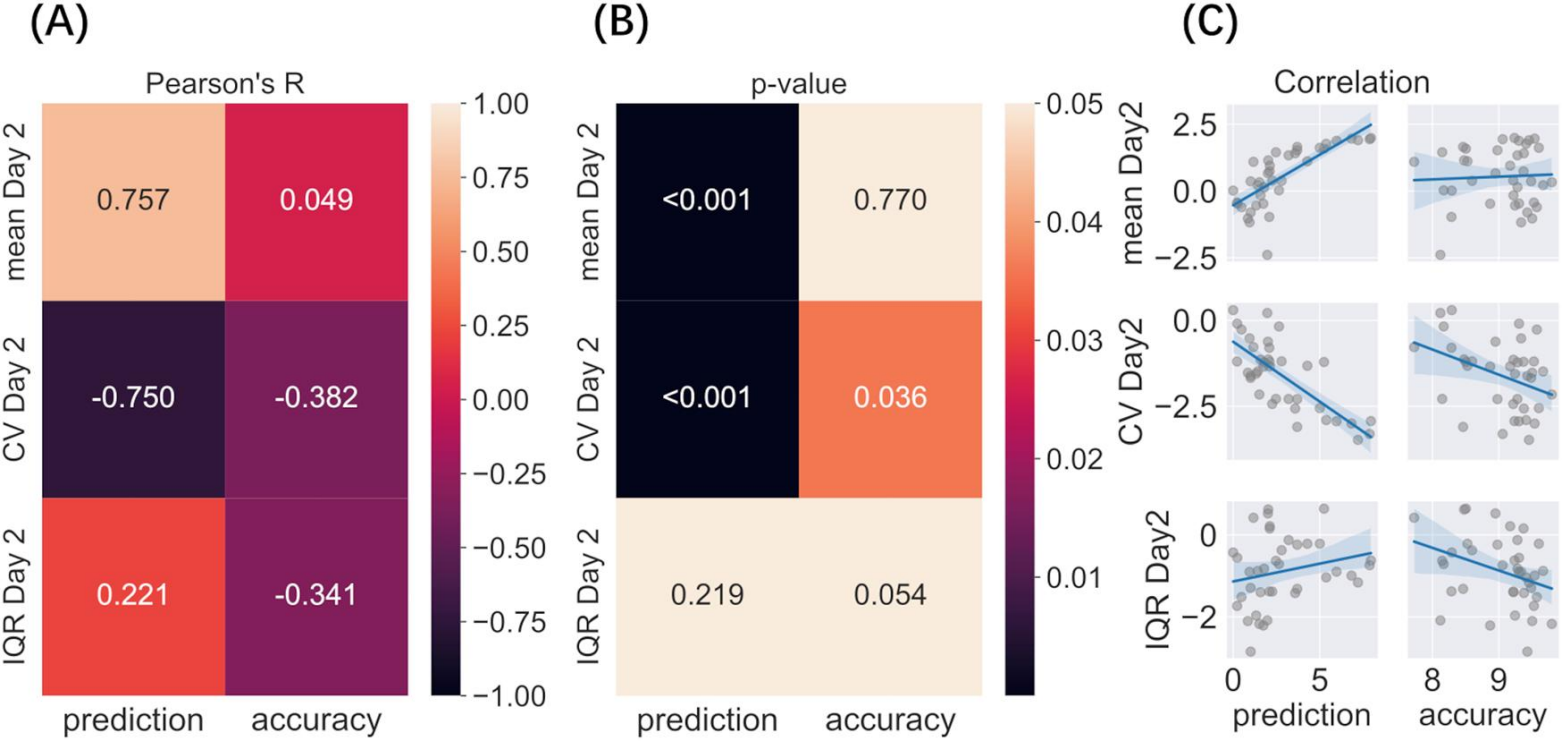
Correlation between predicted and actual pain levels and variability (A–C). Variability factors include log transformed Mean, CV, and IQR. **(A)** Pearson’s correlation among predicted pain on day 1, prediction accuracy (RMSE), mean pain, and variability rated on day 2; **(B)** P-value of each correlation after multiple comparisons; **(C)** Regression of each correlation.

Immediately after completing the pain rating task on day 1, participants predicted how much pain they would experience the following day. The predicted pain intensity was strongly correlated with the mean pain intensity rated on day 2 and was negatively related to its variability (CV, day 2) (Figure 5, Supplementary Table S2). Furthermore, the precision of this prediction, as quantified by RMSE scores, was negatively, although weakly, correlated with the variability (CV) of pain rated on day 2, suggesting that the participants who gave the most accurate predictions may also have experienced less variable pain the following day.

## 4 Discussion

This study offers new insights into the short-term variability of ongoing chronic musculoskeletal pain and its potential clinical relevance. While most research has focused on pain dynamics over days or weeks, our findings highlight that meaningful patterns also emerge over much shorter time scales—on the order of seconds to minutes. We observed strong short-term dependencies in pain ratings, reflected in high lag-1s autocorrelation values and a gradual decay in influence over time. These temporal patterns were consistent across consecutive days, reinforcing the stability of pain dynamics at fine-grained timescales. Moreover, high test-retest reliability for mean pain levels, coefficient of variation (CV), and interquartile range (IQR) suggests that short-term fluctuations are not random noise, but instead follow an individual-specific and structured pattern. Pain variability (CV) was inversely related to mean pain levels, indicating that individuals reporting higher average pain experienced less fluctuation—potentially reflecting more entrenched or rigid pain states. In contrast, the positive correlation between mean pain and IQR suggests that even when pain is severe, moment-to-moment ratings can still vary substantially, highlighting a nuanced and multidimensional picture of pain experience.

Our findings build on and extend prior work on the temporal dynamics of chronic pain, which has traditionally emphasized longer time frames—ranging from days to months—to capture flare-ups, diurnal trends, or treatment responses (1, 4–6, 30). These longer-term patterns have been linked to increased disability, emotional distress, and maladaptive behaviors such as overactivity during pain-free intervals (31–33). However, far less attention has been paid to short-term pain dynamics (8), despite their potential to reveal fast-acting regulatory processes and to better reflect the real-time burden of chronic pain in daily life.

Studying short-term variability is both practical and meaningful. It can be easily measured using brief, app-based self-report tools, and offers a complementary perspective to long-term monitoring. The current study was developed with input from people living with chronic pain, through Patient and Public Engagement activities that emphasized the importance of capturing the unpredictability and moment-to-moment changes in pain. These temporal features are often invisible in traditional assessments, yet they shape how individuals manage their pain and interact with their environments (2–4).

Previous research on short-term variability has largely focused on experimental pain paradigms, where individuals with chronic pain show greater trial-to-trial variability in response to painful stimuli than healthy controls (9). This variability has been linked to prior expectations (17, 34–36), psychological traits such as pain catastrophizing (9), and cognitive load (37), suggesting that short-term fluctuations reflect the interaction of neurophysiological and psychological systems.

Here, we extend these findings to naturalistic, self-reported pain in daily life. We found that the CV of continuous pain ratings was negatively correlated with clinical severity (e.g., BPI severity) and positively associated with self-reported musculoskeletal health (MSK-HQ). In other words, individuals with more severe, persistent pain showed less moment-to-moment variability, whereas those with milder symptoms experienced more fluctuation. This pattern cannot be easily explained by a ceiling effect, as the ratings did not cluster near the top of the scale. Instead, it may reflect the biological stabilization of pain due to central sensitization or impaired descending modulation (38), leading to pain that is both elevated and resistant to short-term modulation.

The observed relationship between short-term variability and clinical outcomes underscores its potential value as a marker of pain severity and flexibility. These findings suggest that high-resolution self-report measures could be developed into practical Patient-Reported Outcome Measures (PROMs), offering insights not only into how much pain a person feels, but how that pain behaves over time. Such metrics could support individualized treatment planning and better reflect patients’ lived experiences. Future studies should examine the stability of these short-term patterns over longer durations (weeks or months), their sensitivity to therapeutic interventions, and their potential for guiding personalized care. All code for implementing the continuous rating task is openly available on Zenodo (39), enabling replication and further development by the research and clinical communities.

In addition, our findings provide insight into the capacity of individuals with chronic pain to anticipate their future pain levels. The strong correlation between participants’ predicted pain on Day 1 and their actual pain ratings on Day 2 suggests that, despite the inherent fluctuations in chronic pain, individuals were able to form reasonably accurate expectations about their near-future experiences. This supports the notion of temporal statistical learning, whereby the brain detects regularities within autocorrelated pain signals to generate predictions about what will come next (16, 17). These results are consistent with broader research on pain expectancy, which shows that anticipatory processes can shape subsequent pain perception and are influenced by contextual factors such as sleep quality and emotional state (40, 41). Together, these findings highlight that the subjective predictability of pain (often dismissed as erratic) may in fact be grounded in learned, temporally structured patterns that individuals can implicitly access and use to guide behavior and coping.

### 4.1 Limitations

Several limitations should be acknowledged. First, the sample was recruited online through the Prolific platform, which may limit the generalizability of the findings. Participants were self-selected and may not fully represent the broader chronic pain population, particularly in terms of age, socioeconomic status, or clinical diagnoses. The relatively small number of male participants who completed both days (*n* = 9) further restricts the representativeness of the sample and may limit the ability to generalize results across sexes.

Second, although efforts were made to ensure data quality (such as attention checks and data pre processing), participant dropout between days was relatively high. This may be due in part to the requirement that Day 2 participation occur at a similar time as Day 1, as well as the low-engagement nature of the task. As a result, analyses involving test-retest reliability were based on a smaller subset of participants, which may reduce statistical power and introduce potential bias.

Third, while continuous pain ratings offer a rich and ecologically valid snapshot of moment-to-moment experience, they still rely on subjective self-report and may be influenced by individual differences in interpretation or interaction with the rating scale. Furthermore, we did not collect physiological data that could complement and validate the self-reported fluctuations in pain.

Finally, the sample covered a relatively wide age range. Although this diversity reflects the broader chronic pain population, the data were not analyzed by age groups (for example, younger adults vs. middle-aged adults). As a result, potential age-related differences in pain variability could not be examined. Moreover, the study focused on short-term variability over two consecutive days; future research is needed to examine how these patterns evolve over longer timescales and in response to treatment or other contextual changes.

Despite these limitations, the study lays important groundwork for exploring short-term variability as a clinically relevant marker and demonstrates the feasibility of remote, high-frequency pain tracking in real-world settings.

## 5 Conclusion

In conclusion, this study underscores the clinical relevance of short-term variability in chronic musculoskeletal pain, revealing that these moment-to-moment fluctuations are not only stable and individually consistent, but also meaningfully linked to pain severity and self-perceived health. Understanding the temporal dynamics of pain at fine-grained timescales could open new avenues for personalized pain management, offering insights into an individual’s regulatory capacity and potential treatment responsiveness. Importantly, the continuous self-report measures used in this study were simple, scalable, and well-suited to remote delivery—highlighting their promise as practical tools for digital health monitoring and individualized assessment in clinical settings.

## Data Availability

The datasets presented in this study can be found in online repositories. The names of the repository/repositories and accession number(s) can be found in the article/Supplementary Material.
