# Supplementary material for: Short-term variability of chronic musculoskeletal pain
Source: Front Pain Res (Lausanne). 2025 Sep 11;6:1626589. doi: 10.3389/fpain.2025.1626589 (PMC12460470; doi:10.3389/fpain.2025.1626589)
Supplement: Supplementary file 1 [file Datasheet1.pdf]

Supplementary Material

1 FLOW CHART OF STUDY PARTICIPANT INCLUSION AND EXCLUSION

Figure S1 illustrates the flow chart detailing the inclusion and exclusion applied to study participants.

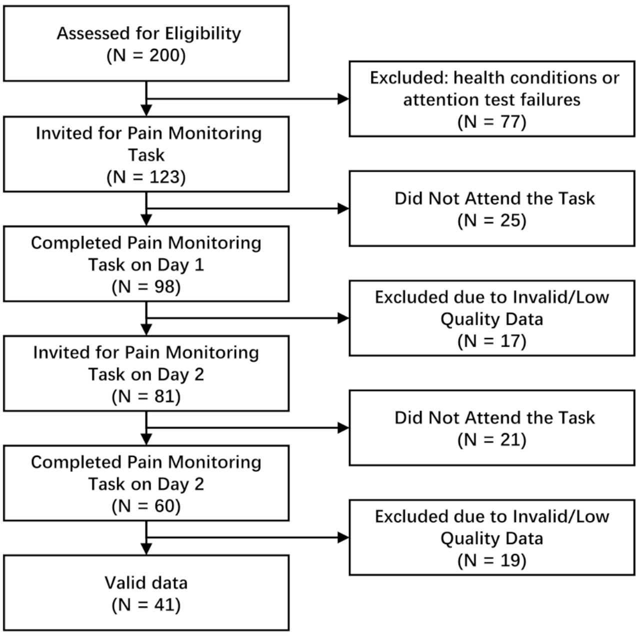

Figure S1. Flow Chart of Study Participant Inclusion and Exclusion. This flowchart illustrates the recruitment and screening process for the study. Of 200 participants recruited, 123 met the inclusion criteria based on health screening and attention checks. After Day 1, 81 participants completed the task, and 41 completed the two-day experiment, with exclusions due to non-attendance or invalid/low-quality data. Invalid or low-quality data were defined as cases where participants failed attention checks or failed to keep their finger on the screen for more than 10 seconds during the rating periods

2 VARIABILITY ANALYSES

Figure S2 presents the distribution of variability factors.

Table S1 summarizes the correlations between mean pain levels, variability measures (CV, IQR), and clinical scores to explore their clinical significance.

Table S2 presents the correlations between variability factors and prediction accuracy.

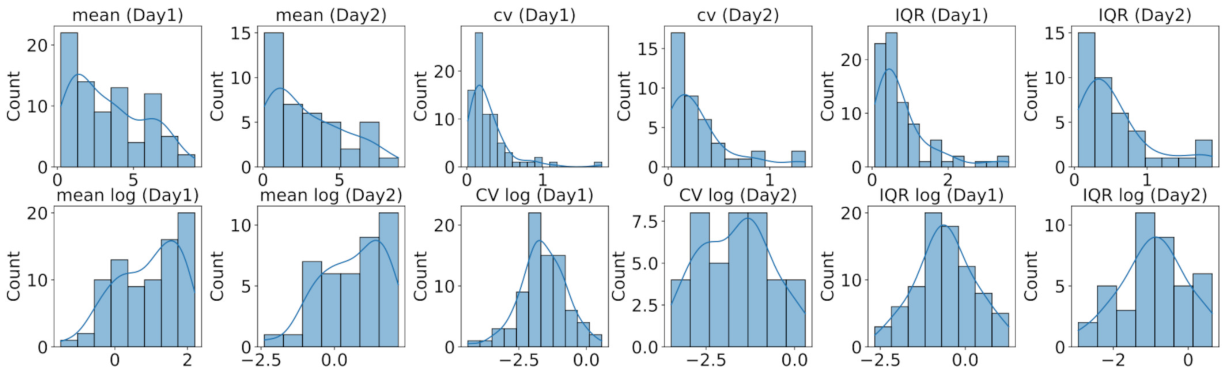

Figure S2. Distribution of Variability Factors Before and After Log Transformation. Distribution of variability factors (Mean, CV, IQR) for both Day 1 and Day 2 is displayed here. The first row represents the distribution based on raw data, while the second row depicts the distribution after log transformation. The distribution is less skewed after the log transformation.

Table S1. Significant Correlation between Variability Factors and Clinical Outcome (mean, CV, IQR v.s. MSK-HQ, BPI severity, PCS)

		Pearson's R	Correlation	p-value
Mean (Day 1)	MSK-HQ	-0.645	strong negative	$<$ 0.001
Mean (Day 1)	BPI Severity	0.752	strong positive	$<$ 0.001
Mean (Day 1)	PCS	0.483	moderate positive	0.004
CV (Day 1)	MSK-HQ	0.530	strong positive	0.002
CV (Day 1)	BPI Severity	-0.609	strong negative	$<$ 0.001
IQR (Day 1)	BPI Severity	0.220		0.109
IQR (Day 1)	PCS	0.299		0.061
Mean (Day 2)	MSK-HQ	-0.595	strong negative	$<$ 0.001
Mean (Day 2)	BPI Severity	0.742	strong positive	$<$ 0.001
Mean (Day 2)	PCS	0.537	strong positive	0.001
CV (Day 2)	MSK-HQ	0.485	moderate positive	0.002
CV (Day 2)	BPI Severity	-0.634	strong negative	$<$ 0.001
IQR (Day 2)	BPI Severity	0.380	moderate positive	0.008
IQR (Day 2)	PCS	0.445	moderate positive	0.003

Table S2. Significant Correlation between Variability Factors and Prediction Accuracy (mean, CV, IQR v.s. prediction and accuracy)

		Pearson's R	Correlation	p-value
prediction	Mean	0.757	strong positive	$<$ 0.001
prediction	CV	-0.750	strong negative	$<$ 0.001
Accuracy	CV	-0.382	moderate negative	0.036

3 SCREENING QUESTIONNAIRE

1. How old are you?
2. What is your gender at birth?
   - Male
   - Female
   - Prefer not to say
3. How long have you had pain to your **back** for?

- I don't have back pain
- < 1 month
- 1-3 months
- 3-6 months
- 6-12 months
- 1-2 years
- 2-5 years
- 5-10 years
- 10-20 years
- > 20 years

4. How long have you had pain to your **neck** for?

- I don't have back pain
- < 1 month
- 1-3 months
- 3-6 months
- 6-12 months
- 1-2 years
- 2-5 years
- 5-10 years
- 10-20 years
- > 20 years

5. How long have you had pain to your **legs, knees or feet** for?

- I don't have back pain
- < 1 month
- 1-3 months
- 3-6 months
- 6-12 months
- 1-2 years
- 2-5 years
- 5-10 years
- 10-20 years
- > 20 years

6. How long have you had pain to your **arms or hands** for?

- I don't have back pain
- < 1 month
- 1-3 months
- 3-6 months
- 6-12 months
- 1-2 years
- 2-5 years
- 5-10 years
- 10-20 years
- > 20 years

7. Do you identify as currently suffering from any of the following pain conditions? Please tick all that apply

- Prefer not to say
- Abdominal pain/IBS
- Arthritis (inflammatory: rheumatoid, psoriatic, etc)
- Arthritis (non-inflammatory, such as osteoarthritis)
- Complex regional pain syndrome
- Fibromyalgia
- Headache/Migraine
- Multiple Sclerosis
- Neuropathy
- Pelvic pain
- Endometriosis
- Trigeminal neuralgia
- Spinal cord injury
- stroke pain

- Other pain condition (if so, please specify below:) ______

8. Have you received a diagnosis for any of these conditions? If Yes, please provide the approximate dates of diagnoses of each condition.

- No
- Yes
  Details ________

9. What treatment (pharmacological, therapy, etc), if any, are you taking for your conditions(s)? Please give detail about treatment and time periods for treatment if possible.
   - ________________
10. Do you identify as living with, or have you received a diagnosis of anyother medical condition?
    - No
    - Yes
      If yes, please specify:
      Details of conditions(s) and date(s) of onset and/or diagnosis:________
11. Do you experience any mental health difficulty? If so, how long you have experienced it, have you been diagnosed, and do you take any treatment for it? Please also provide any other details.
    - ________________
12. Have you ever had or are experiencing one of the following conditions? Please tick all that apply.
    - Amnesia (a memory difficulty)
    - Attention deficit hyperactivity disorder (ADHD)
    - Brain cancer
    - Brain haemorrhage
    - Brain injury
    - Cognitive impairment (e.g., memory, language, attention etc)
    - Cognitive developmental delay
    - Dementia
    - Dyslexia
    - Epilepsy
    - Hydrocephalus
    - Learning disorder
    - Neurodegenerative disorder
    - Stroke
    - Other neurological condition: Please specify: ______
    - Other cognitive condition: Please specify: ______

4 PARTICIPANT-SPECIFIC CLINICAL DETAILS VIA MSK-HQ, BPI AND PCS QUESTIONNAIRE.

	Age	Sex	Pain Duration (year)	Type of Pain	Medical Diagnosis (If Any)
1	40	Female	15.00	back pain	No
2	30	Female	3.50	back pain	No
3	49	Female	7.50	back pain	Trigeminal neuralgia (diagnosed 2010)
4	31	Female	3.50	neck pain	No
5	54	Female	20.00	back pain	Diagnosed 2022
6	57	Female	7.50	back pain	Lumbar disc herniation(sciatic nerve)
7	22	Female	3.50	back pain	No
8	37	Male	3.50	back pain	No
9	35	Female	3.50	back pain	Irritable bowel syndrome (IBS)
10	29	Female	3.50	back pain	No
11	32	Female	15.00	back pain	No
12	37	Female	15.00	back pain	No
13	42	Female	3.50	back pain	No
14	61	Male	7.50	legs, knees or feet	No
15	38	Female	3.50	back pain	No
16	52	Female	20.00	back pain	Diagnosed Jan 2022
17	48	Female	15.00	back pain	No
18	58	Female	1.50	legs, knees or feet	No
19	32	Male	15.00	back pain	No
20	43	Female	0.75	legs, knees or feet	No
21	36	Female	7.50	legs, knees or feet	Intraosseous cyst (ankle)
22	52	Female	15.00	back pain	Diagnosed (unspecified condition)
23	27	Male	3.50	back pain	No
24	65	Female	3.50	back pain	IBS(1985), Osteoparthrtis(2012)
25	58	Female	20.00	back pain	Diagnosed 1992
26	54	Female	3.50	arms or hands	No
27	38	Male	7.50	back pain	No
28	46	Male	20.00	neck pain	No
29	41	Male	7.50	legs, knees or feet	No
30	50	Female	3.50	legs, knees or feet	endometriosis(2021), arthritis(2020)
31	45	Female	7.50	back pain	No
32	53	Female	15.00	legs, knees or feet	No
33	62	Male	3.50	legs, knees or feet	Diagnosed 2022
34	41	Female	7.50	back pain	No
35	54	Female	20.00	back pain	OI(birth), migraine(1990), arthritis(1988)
36	28	Female	3.50	back pain	No
37	36	Female	15.00	legs, knees or feet	DVT(2008), post-thrombotic syndrome(2020)
38	51	Male	1.50	back pain	No
39	63	Female	7.50	legs, knees or feet	Fibromyalgia, arthritis, Achilles tendinitis
40	43	Female	7.50	legs, knees or feet	Diagnosed 2016

	Age	Sex	Pain Duration (year)	Type of Pain	Medical Diagnosis (If Any)
41	53	Female	3.50	arms or hands	No
42	38	Female	15.00	back pain	No
43	30	Male	0.75	back pain	No
44	50	Male	20.00	back pain	No
45	44	Female	3.50	back pain	diagnosed 2023; symptoms since 2017
46	45	Female	15.00	back pain	IBS(20 years), chest pain
47	36	Female	0.75	back pain	No
48	60	Female	20.00	back pain	Diagnosed 2000
49	54	Female	7.50	arms or hands	Diagnosed over 10 years
50	62	Male	20.00	neck pain	No
51	52	Male	15.00	legs, knees or feet	Diagnosed 2015
52	64	Male	20.00	back pain	Diagnosed 2010
53	25	Female	7.50	back pain	Diagnosed (unspecified condition)
54	24	Male	1.50	back pain	Diagnosed 2021
55	37	Female	15.00	back pain	No
56	30	Female	7.50	back pain	Migraine(2018)
57	45	Male	3.50	legs, knees or feet	No
58	64	Male	20.00	neck pain	IBS diagnosed 2020; symptoms much longer
59	62	Female	20.00	neck pain	2004
60	61	Female	15.00	back pain	Disc herniation(lumbar, cervical), suspected RA
61	40	Male	15.00	back pain	Lumbar herniation; shoulder lesion arthritis
62	50	Female	3.50	back pain	No
63	57	Male	7.50	back pain	No
64	38	Male	7.50	back pain	No
65	47	Female	7.50	back pain	Lower back injury (2016), diagnosed 2018
66	58	Female	20.00	back pain	CBP(1980), IBS(2000), diverticulitis(2000s)
67	64	Female	3.50	back pain	Diagnosed 2019
68	42	Female	3.50	legs, knees or feet	No
69	36	Male	7.50	back pain	No
70	33	Female	1.50	back pain	No
71	29	Female	15.00	back pain	Diagnosed 2008
72	55	Male	20.00	back pain	Diagnosed 2008
73	56	Female	15.00	back pain	Osteoarthritis(2013)
74	48	Female	3.50	back pain	Diagnosed 2021
75	26	Female	3.50	back pain	No
76	52	Female	20.00	legs, knees or feet	ME (1995)
77	36	Male	7.50	back pain	Diagnosed 2020
78	51	Female	20.00	back pain	Diagnosed 2003
79	63	Female	3.50	legs, knees or feet	Knee arthritis (2021, bilateral)
80	42	Female	15.00	back pain	endometriosis(1999), calcification(2022)
81	61	Female	3.50	legs, knees or feet	No

	Pain Medication Taken (If Any)
1	OTC pain relievers
2	none
3	Pregabalin
4	Pain relievers
5	Amitriptyline 30 mg daily; tramadol (occasional use)
6	Nortriptyline, nightly
7	Ibuprofen as needed
8	Pain relievers
9	Dietary modifications and antacids for IBS; physical therapy and exercise for back pain
10	None
11	Pain relievers as needed; prefers stretching exercises
12	Pain relievers (occasional use)
13	Physiotherapy: 1 session/month; 3 sessions received to date
14	Non-prescription anti-inflammatory tablets; arm/hand exercises
15	
16	Chiropractor for 6 months; Physiotherapy for 2 months
17	Sports Physio for back pain
18	paracetamol, ibuprofen, as needed
19	No formal treatment; exercises to strengthen back muscles
20	Pain relievers
21	
22	Amitriptyline for nerve pain, paracetamol and ibuprofen
23	Physiotherapeutic exercises
24	Monthly massage; occasional Reiki; regular exercise; topical natural oil gel for joints
25	Aspirin, paracetamol, and topical anti-inflammatory gel for back and shoulder pain
26	Vitamins and mineral supplements; exercise; heat therapy
27	
28	none
29	Stretching, yoga, walking
30	Progesterone for endometriosis; non-opioid analgesics for hip and knee pain
31	
32	Co-codamol and paracetamol; naproxen discontinued due to side effects
33	Pain relievers
34	Codeine (as needed)
35	Morphine, Gabapentin, Amitriptyline
36	none
37	Blood thinners and compression tights
38	Ibuprofen as needed; physiotherapy and chiropractic treatment
39	codeine, Amitriptyline and Citalopram
40	Methotrexate injections (weekly)

	Pain Medication Taken (If Any)
41	Pain relievers and anti-inflammatory medications
42	Chiropractor every 6 weeks
43	
44	Back massage 2-3 times/year
45	Sertraline for pain management
46	Lansoprazole
47	
48	simple pain relievers like paracetamol
49	Metamucil(IBS); massage, physiotherapy, and extra-strength Tylenol(shoulder pain); shoulder surgery
50	none
51	Meloxicam tablets; meditation; home heat therapy
52	Statins and aspirin
53	Pain relievers as needed, used intermittently since onset of pain
54	Propranolol
55	
56	Prescribed medication
57	Regular stretching and exercise
58	OTC remedies (charcoal, probiotics, gas relief)
59	Discontinued Celebrex; currently uses muscle relaxants as needed and occasional ibuprofen
60	Previously used strong pain meds; now rarely uses OTC analgesics
61	Naproxen and omeprazole
62	Physiotherapy (past)
63	Paracetamol
64	Ibuprofen as needed
65	PRN analgesics (paracetamol, ibuprofen); physiotherapy, stretching, and yoga several times/week
66	Co-codamol (intermittent), mirtazapine, amitriptyline (long-term)
67	Steroids
68	OTC pain relievers
69	Regular physiotherapy
70	Massage therapy
71	Physiotherapy and chiropractic treatment
72	Humira, methotrexate, tramadol
73	Steroid injections (2015–2018); dihydrocodeine, paracetamol, gabapentin (ongoing)
74	Anti-inflammatories as needed
75	Pain medication (ibuprofen), no other treatment
76	
77	Spinal injections
78	Medication and exercise
79	Physiotherapy (initial treatment)
80	pain relievers
81	OTC pain relievers

	MSK-HQ	BPI Severity	BPI Interference	PCS Rumination	PCS Magnification	PCS Helplessness
1	43	4.50	2.57			
2	43	3.25	3.00	12.00	3.00	3.00
3	22			4.00	3.00	7.00
4	38	2.00	1.00	4.00	2.00	5.00
5	27	4.50	3.57	8.00	3.50	9.50
6	35	3.25	8.29			
7	33	5.00	6.14	4.00	5.00	8.00
8	44					
9	36	6.50	4.71	2.00	1.00	1.00
10	41			3.00	2.00	2.00
11	22	6.75	7.43	7.00	7.00	10.00
12	44	2.00	1.29	2.00	3.00	2.00
13	35	4.00	7.29			
14	38			6.00	3.00	9.00
15	35	3.75	3.00	5.00	3.00	7.00
16	37	3.75	4.43	10.00	9.00	12.00
17	39			5.00	2.00	4.00
18	44	1.50	0.29	0.00	0.00	0.00
19	43	2.50	0.71	0.00	2.00	2.00
20	44	5.00	1.43	3.00	1.00	4.00
21	43	1.25	0.14	0.00	2.00	0.00
22	45			5.00	5.00	7.00
23	40	2.25	0.43	1.00	0.00	1.00
24	48	2.25	1.71	3.00	0.00	1.00
25	43	6.25	2.00	6.00		2.00
26	50	4.50	4.14	1.00	1.00	2.00
27	40	2.75	3.00	5.00	3.00	8.00
28	36	5.25	6.71	4.00	5.00	9.00
29	42	5.25	5.14	4.00	1.00	5.00
30	24	3.75	4.86	12.00	7.00	14.00
31	24	4.00	4.71	6.00	5.00	7.00
32	14	5.75	7.29	12.00	9.00	14.00
33	34	5.00	4.86	5.00	4.00	4.00
34	50	2.50	2.86	0.00	1.00	0.00
35	21			4.00	2.00	5.00
36	41	2.75	2.43			
37	35	2.25	2.14	3.00	3.00	1.00
38	42	2.75	0.57			
39	32	4.25	5.86	3.00	2.00	6.00
40	31	4.00	4.71	6.00	5.00	7.00

	MSK-HQ	BPI Severity	BPI Interference	PCS Rumination	PCS Magnification	PCS Helplessness
41	24	6.50	5.43	10.00	4.00	12.00
42	46			2.00	1.00	1.00
43	45	1.25	0.29	0.00	1.00	1.00
44	26	2.00	2.86			
45	26	6.75	6.43	12.00	12.00	14.00
46	34					
47	45	1.00	0.00	0.00	1.00	0.00
48	45	2.25	0.29	4.00	4.00	4.00
49	17	6.50	4.00	6.00	5.00	13.00
50	47					
51	28	4.75	6.86	9.00	7.00	13.00
52	38	2.25	1.71	11.00	0.00	7.00
53	32	3.25	2.00	7.00	4.00	8.00
54	33	7.00	3.71	8.00	8.00	6.00
55	29	1.00	0.00	0.00	0.00	0.00
56	40	2.50	1.71	3.00	1.00	3.00
57	40	3.00	0.71	0.00	1.00	2.00
58	50	2.50	1.14	5.00	1.00	3.00
59	40	5.25	1.43			
60	38	4.50	1.29	0.00	3.00	2.00
61	38	4.50	2.29	1.00	2.00	3.00
62	48	1.00	0.43	0.00	0.00	1.00
63	50	2.00	1.71	2.00	0.00	
64	42	1.75	2.29	2.00	1.00	1.00
65	40	4.75	2.57			
66	21	7.00	7.43	6.00	3.00	8.00
67	46	3.25	3.14			
68	42	2.00	0.57	4.00	2.00	2.00
69	38	3.00	5.29	4.00	5.00	6.00
70	44	3.00	2.86	8.00	4.00	3.00
71	35			9.50	4.50	10.00
72	9	4.25	7.57	15.00	8.00	21.00
73	16	8.00	9.00	13.00	11.00	
74	37	4.25	6.00	10.00	5.00	13.00
75	42	5.25	4.57	3.00	1.00	5.00
76	45	4.50	5.43	2.00	1.00	5.00
77	41	4.50	1.86	4.00	2.00	7.00
78	31			7.00	1.00	7.00
79	36	2.75	2.71			
80	29	4.75	5.00	6.00	4.00	6.00
81	35	2.75	2.29			
