# Supplementary figures and images for: Short-term variability of chronic musculoskeletal pain
Source: Front Pain Res (Lausanne). 2025 Sep 11;6:1626589. doi: 10.3389/fpain.2025.1626589 (PMC12460470; doi:10.3389/fpain.2025.1626589)

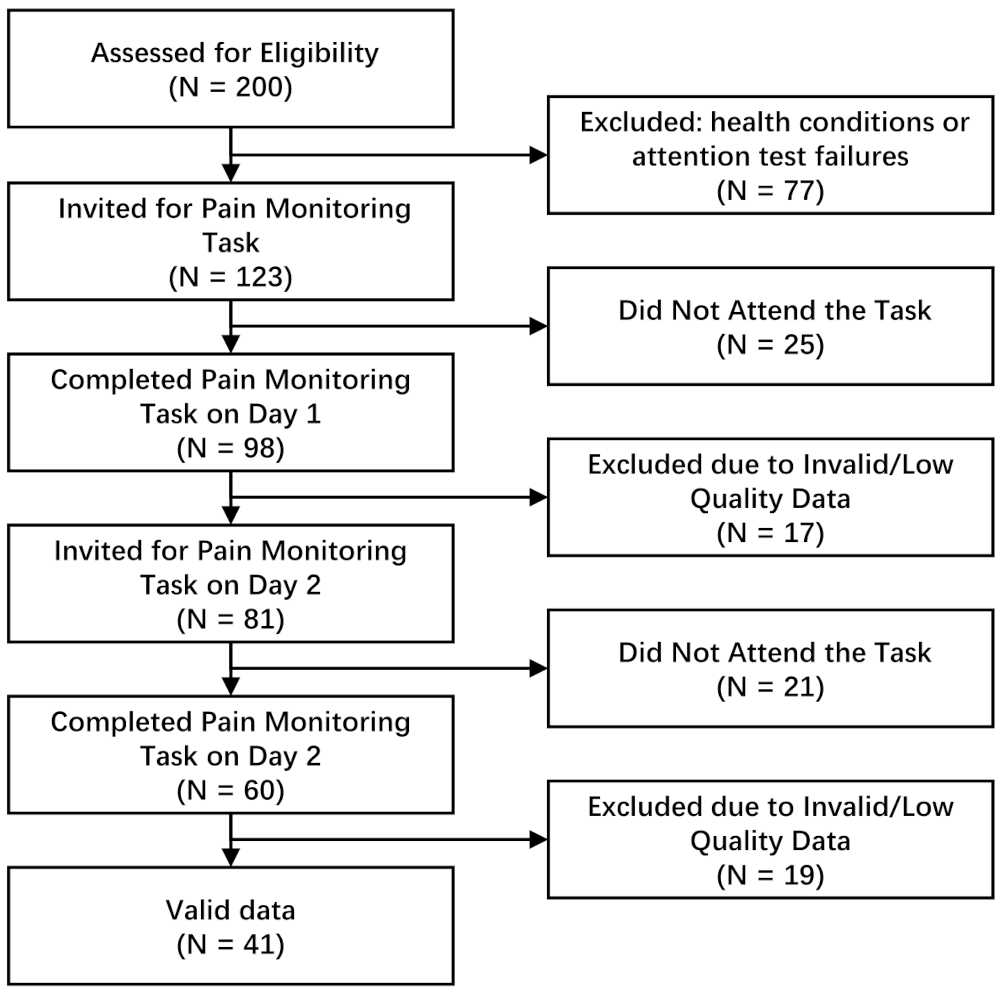

Supplement: Supplementary file 2 [file Image1.tif]

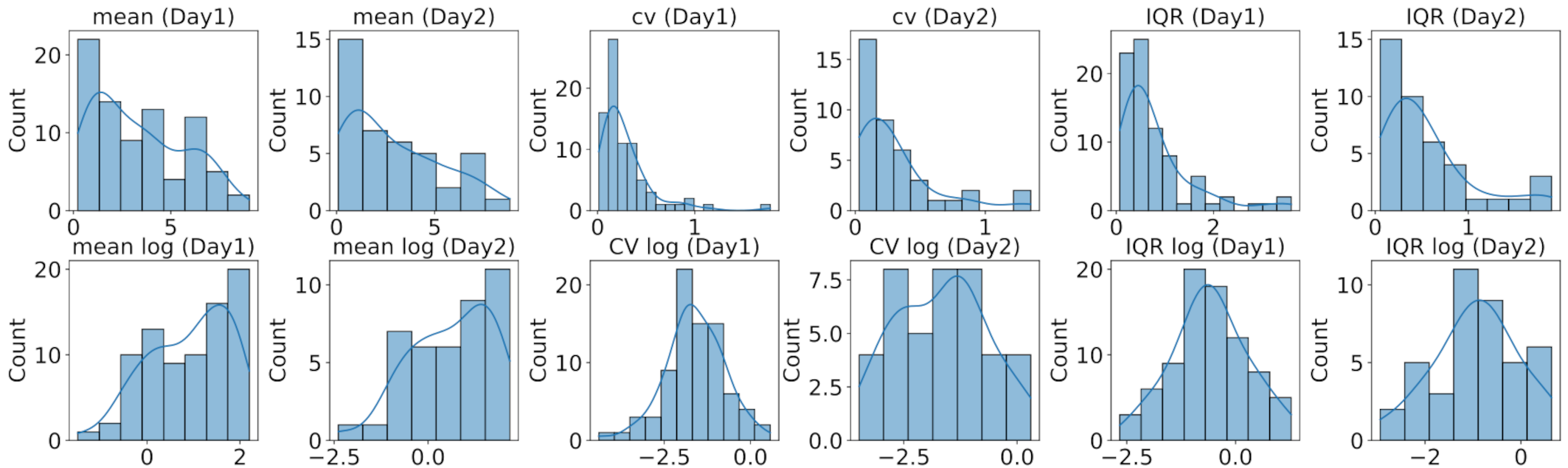

Supplement: Supplementary file 3 [file Image2.tif]
